# Inadvertent portal vein stenting during endoscopic retrograde cholangiopancreatography for distal bile duct cancer: endoscopic rescue and spontaneous resolution of thrombosis

**DOI:** 10.1055/a-2619-1220

**Published:** 2025-07-01

**Authors:** Seong-Hun Kim, Hyung Ku Chon

**Affiliations:** 1 Division of Gastroenterology, Department of Internal Medicine, Jeonbuk National University Medical School, Jeonju, Korea; 2 Division of Biliopancreas, Department of Internal Medicine, Wonkwang University College of Medicine, Iksan, Korea; 3 Institute of Wonkwang Medical Science, Iksan, Korea

An 86-year-old woman with distal bile duct cancer and obstructive cholangitis presented with jaundice and a 5-kg weight loss over 1 month. She underwent endoscopic retrograde cholangiopancreatography (ERCP) for biliary decompression ( Fig. 1 ). Initial selective biliary cannulation failed, prompting placement of a plastic pancreatic duct stent followed by transpancreatic septotomy. A guidewire was advanced in the presumed direction of the bile duct and contrast injection suggested biliary opacification. Assuming correct guidewire placement, a fully covered self-expandable metal stent (FCSEMS) (HANAROSTENT Biliary Full Cover Benefit, 8 mm × 6 cm; M.I. Tech, Pyeongtaek, South Korea) was deployed. Substantial resistance was encountered during stent deployment, and the contrast rapidly washed out, raising suspicion of extrabiliary placement. A contrast-enhanced computed tomography (CECT) performed 2 hours later revealed that the FCSEMS had traversed the distal bile duct mass and had been inadvertently placed in the portal vein, resulting in acute portal vein thrombosis ( Fig. 2 ).

**Fig. 1:**
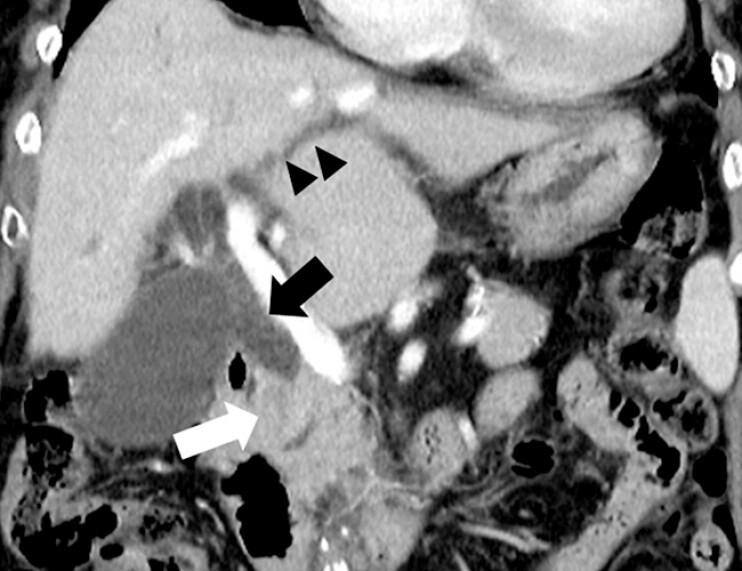
Coronal contrast-enhanced computed tomography images showing an enhancing mass (white arrow) in the distal common bile duct with upstream dilation of the intrahepatic (black arrow heads) and extrahepatic bile ducts (black arrow), suggestive of distal bile duct cancer.

**Fig. 2:**
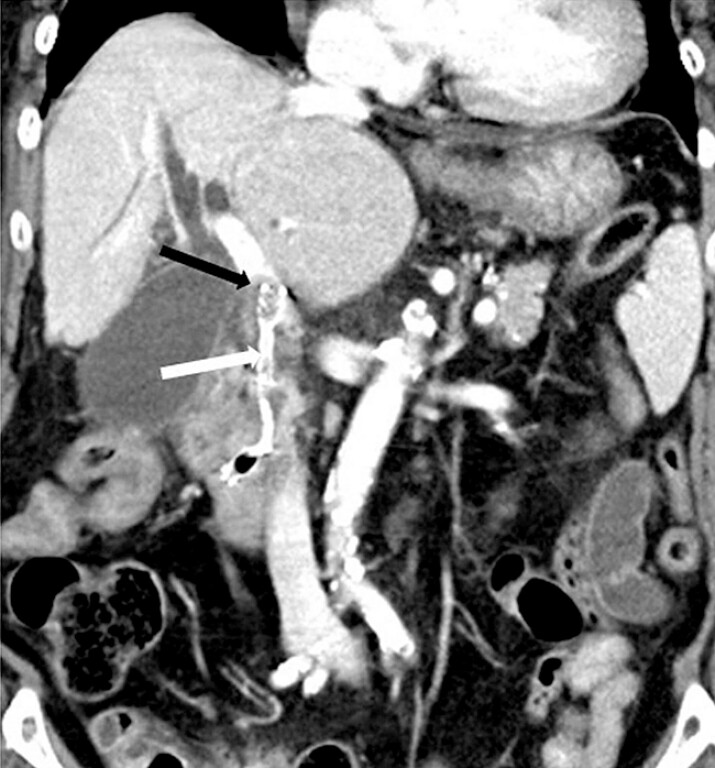
Contrast-enhanced computed tomography obtained 2 hours post-endoscopic retrograde cholangiopancreatography showing maldeployed fully covered self-expandable metal stent (white arrow) in the portal vein via the distal bile duct mass, with associated portal vein thrombosis (black arrow).

An emergency ERCP was performed, during which selective biliary cannulation was achieved via needle-knife precutting. The misplaced stent was retrieved, and a new FCSEMS (HANAROSTENT Biliary Lasso Full covered, 10 mm × 6 cm; M.I. Tech) and plastic stents were successfully deployed into the bile duct ( Fig. 3 , Video 1 ). The patient recovered uneventfully and was discharged 10 days later. Follow-up CECT 7 days later showed portal vein thrombosis, which had resolved spontaneously by Day 153 without anticoagulation ( Fig. 4 ).

**Fig. 3:**
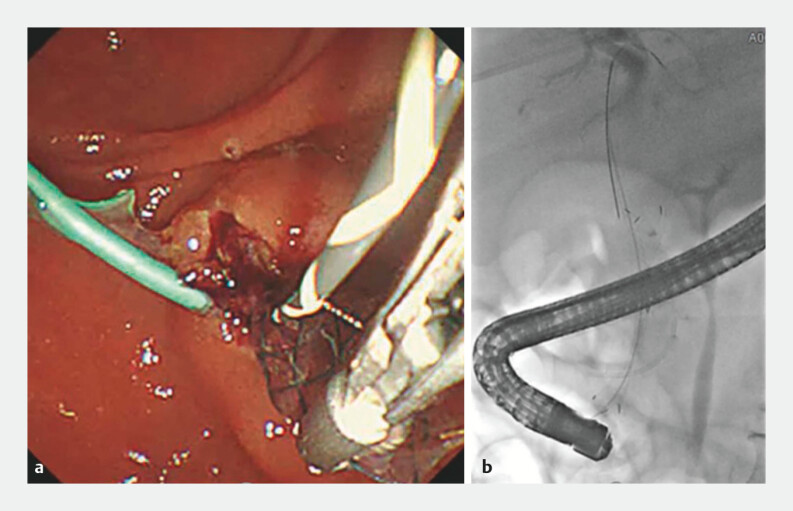
Retrieval and replacement of the fully covered self-expandable metal stent (FCSEMS). **a** Endoscopic image showing selective bile duct cannulation with subsequent retrieval of the maldeployed FCSEMS using forceps during emergency endoscopic retrograde cholangiopancreatography. **b** Fluoroscopic image demonstrating correct placement of a new FCSEMS within the bile duct.

Endoscopic rescue and spontaneous resolution of thrombosis after inadvertent portal vein stenting during endoscopic retrograde cholangiopancreatography for distal bile duct cancer.Video 1

**Fig. 4:**
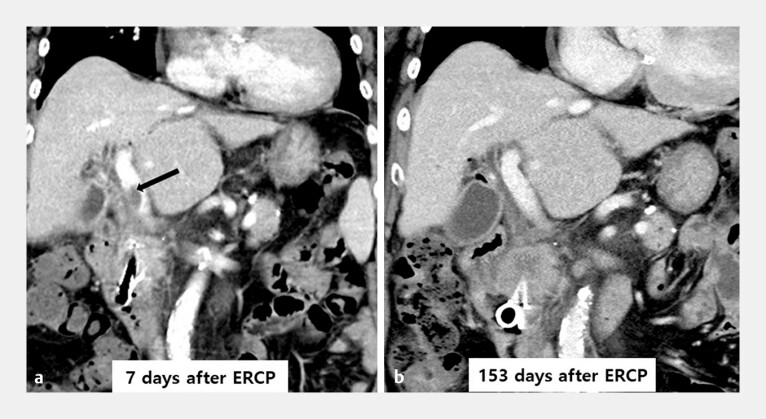
Contrast-enhanced computed tomography. **a** At 7 days post- endoscopic retrograde cholangiopancreatography, demonstrating portal vein thrombosis (black arrow). **b** The portal vein thrombosis had spontaneously resolved by follow-up imaging on Day 153 without anticoagulant therapy.

Portal vein injury is a rare but potentially fatal complication of ERCP 1 2 . This case underscores the importance of recognizing signs of vascular misplacement, such as rapid contrast washout and deployment resistance. Early recognition and prompt endoscopic management are critical to avoiding serious complications. Notably, the spontaneous resolution of portal vein thrombosis without anticoagulation suggests that conservative management may be appropriate in selected patients with localized, nonocclusive thrombosis.

Endoscopy_UCTN_Code_TTT_1AR_2AZ

## Notes

Endoscopy E-Videos https://eref.thieme.de/e-videos
*E-Videos* is an open access online section of the journal *Endoscopy* , reporting on interesting cases and new techniques in gastroenterological endoscopy. All papers include a high-quality video and are published with a Creative Commons CC-BY license. Endoscopy E-Videos qualify for HINARI discounts and waivers and eligibility is automatically checked during the submission process. We grant 100% waivers to articles whose corresponding authors are based in Group A countries and 50% waivers to those who are based in Group B countries as classified by Research4Life (see: https://www.research4life.org/access/eligibility/ ). This section has its own submission website at https://mc.manuscriptcentral.com/e-videos .
